# A systematic review and meta-analysis of school-based interventions with health education to reduce body mass index in adolescents aged 10 to 19 years

**DOI:** 10.1186/s12966-020-01065-9

**Published:** 2021-01-04

**Authors:** Chandni Maria Jacob, Polly Louise Hardy-Johnson, Hazel M. Inskip, Taylor Morris, Camille M. Parsons, Millie Barrett, Mark Hanson, Kathryn Woods-Townsend, Janis Baird

**Affiliations:** 1 Academic Unit of Human Development and Health, Faculty of Medicine, University of Southampton, Southampton, UK; 2 NIHR Southampton Biomedical Research Centre, University Hospital Southampton NHS Foundation Trust, University of Southampton, Southampton, UK; 3 Institute of Developmental Sciences, Faculty of Medicine, Southampton General Hospital, University of Southampton, Mail point 887, Tremona Road, Southampton, SO16 6YD UK; 4 MRC Lifecourse Epidemiology Unit, University of Southampton, Southampton General Hospital, Level two, room 306, Tremona Road, Southampton, SO16 6YD UK; 5 Southampton Education School, Faculty of Social Sciences, University of Southampton, Southampton, UK

**Keywords:** Adolescent health, Body mass index, Obesity, School, Health education, Physical activity, Diet, Nutrition, Intervention

## Abstract

**Background:**

Adolescents are increasingly susceptible to obesity, and thus at risk of later non-communicable diseases, due to changes in food choices, physical activity levels and exposure to an obesogenic environment. This review aimed to synthesize the literature investigating the effectiveness of health education interventions delivered in school settings to prevent overweight and obesity and/ or reduce BMI in adolescents, and to explore the key features of effectiveness.

**Methods:**

A systematic search of electronic databases including MEDLINE, CINAHL, PsychINFO and ERIC for papers published from Jan 2006 was carried out in 2020, following PRISMA guidelines. Studies that evaluated health education interventions in 10–19-year-olds delivered in schools in high-income countries, with a control group and reported BMI/BMI z-score were selected. Three researchers screened titles and abstracts, conducted data extraction and assessed quality of the full text publications. A third of the papers from each set were cross-checked by another reviewer. A meta-analysis of a sub-set of studies was conducted for BMI z-score.

**Results:**

Thirty-three interventions based on 39 publications were included in the review. Most studies evaluated multi-component interventions using health education to improve behaviours related to diet, physical activity and body composition measures. Fourteen interventions were associated with reduced BMI/BMI z-score. Most interventions (*n* = 22) were delivered by teachers in classroom settings, 19 of which trained teachers before the intervention. The multi-component interventions (*n* = 26) included strategies such as environment modifications (*n* = 10), digital interventions (*n* = 15) and parent involvement (*n* = 16). Fourteen studies had a low risk of bias, followed by 10 with medium and nine with a high risk of bias. Fourteen studies were included in a random-effects meta-analysis for BMI z-score. The pooled estimate of this meta-analysis showed a small difference between intervention and control in change in BMI z-score (− 0.06 [95% CI -0.10, − 0.03]). A funnel plot indicated that some degree of publication bias was operating, and hence the effect size might be inflated.

**Conclusions:**

Findings from our review suggest that school-based health education interventions have the public health potential to lower BMI towards a healthier range in adolescents. Multi-component interventions involving key stakeholders such as teachers and parents and digital components are a promising strategy.

**Supplementary Information:**

The online version contains supplementary material available at 10.1186/s12966-020-01065-9.

## Background

Approximately 340 million children and adolescents aged 5–19 years had overweight or obesity globally in 2016 [1]. Almost 80% of adolescents with obesity will have obesity as adults [2] and the prevalence of morbid obesity in adults is higher among those who had obesity as adolescents [3]. Obesity in childhood and adolescence is associated with an increased risk of non-communicable diseases (NCDs) such as Type 2 diabetes, cardiovascular disease, chronic obstructive lung disease and some forms of cancer [4]. Adolescents with excess weight or obesity often have decreased self-esteem and may be subjected to bullying and discrimination, increasing the risk of poor psychological health and eating disorders [5, 6].

Adolescence is a transitional period characterized by critical changes in body composition, insulin sensitivity, health behaviours and psychological and social functioning, as well as increased autonomy [5, 7]. The likelihood of unhealthy eating behaviours, high screen-time, disordered sleep patterns and decreased participation in physical activity (especially among girls) increases during adolescence [8–11]. Factors leading to adolescent obesity can be broadly categorized into individual (food preferences, taste and perceptions, self-efficacy for making healthy choices, and convenience), social (including family and peer relationships), demographic (socioeconomic status (SES)) and environmental (mass media, easy access to fast-food outlets and vending machines, lack of safe active recreation and travel options) factors [12, 13]. It has been suggested that obesity prevention interventions may be more effective in adolescents than younger children, as they are more likely to understand the concepts and have more autonomy, for example about food choices [14].

Previous systematic reviews covering the period before 2006 have evaluated the effectiveness of interventions to improve obesity-related outcomes such as body mass index (BMI), physical activity and dietary behaviours in children and adolescents together, or for particular countries [15–18]. However growing evidence suggests that the school setting provides a platform for effective and sustained intervention delivery to prevent overweight and obesity [19, 20]. Such interventions have often incorporated methods such as health education, providing healthy school meals, parental involvement, and community engagement [15, 21–23]. As adolescents are already engaging in formal education and activities with their peers, schools provide an ideal platform for delivering health interventions, but most reviews examining the effects of school-based programmes on BMI have considered children and adolescents together. A meta-analysis (*n* = 5) showed no significant change in the BMI of children and adolescents receiving the intervention compared with control groups but data disaggregated for 2–19 year-olds were not presented [16]. Fewer trials based in schools were specifically for adolescents, [22] but changing health behaviour patterns may mean that strategies to prevent obesity in this age group require a different approach from those used in younger children.

In the UK, increasing health education in schools has been recommended as part of the government’s Childhood Obesity Strategy [24]. Health education, provided in daytime and after-school programmes, has been widely recommended as a tool to address obesity [25], by encouraging behaviour change and improved health literacy [26]. However, to date, reported effects of health education on body composition and weight have been mixed, possibly due to the short duration of interventions and a lack of attention to accompanying lifestyle changes outside the school environment [27]. The effectiveness of health education as a way of reducing BMI in adolescence has not been reviewed.

### Aim

The aim of this review was to synthesize evidence to answer the following questions: 1) What is the effectiveness of health education interventions delivered in school settings to prevent overweight and obesity and/ or reduce BMI in adolescents? 2) What are the key features of effective interventions?

## Methods

### Search strategy

A comprehensive and systematic search of published literature was undertaken in January 2017, and updated in June 2020, in accordance with the Preferred Reporting Items for Systematic reviews and Meta-Analysis (PRISMA) [28] and the University of York Centre for Reviews and Dissemination (CRD) Guidelines [29]. We focused our searches on the period since 2006 which was not covered by existing high-quality systematic reviews of school-based interventions. The following electronic databases were searched in full: MEDLINE, ERIC, PsychINFO and CINAHL. A combination of medical subject headings (MESH) and free text keywords were used to find intervention studies, limited to English language and published between January 2006 and 2020. Separate search strings were developed for diet (e.g., fruit), physical activity (e.g., exercise, sport) and obesity (e.g., overweight, obese), intervention (e.g., education, health literacy) and adolescents (e.g., teen, youth) (see Supplementary Material 1). We consulted an information specialist (LP) to review and comment on the search strategy and contacted experts in the field to identify studies not located in the database searches. Reference lists of included articles were also screened. The proposal for this review was registered with PROSPERO (ID: CRD42016053477).

### Selection criteria

Experimental studies that investigated the effectiveness of health education interventions in adolescents aged 10–19 years in school settings [30], and which reported BMI and/or BMI z-scores as outcomes [31] were included in this review. Table 1 presents further details on the rationale for the inclusion criteria. Included studies needed to have a control or comparison group and pre/post-intervention measures for BMI outcomes (at least one post-intervention measure). BMI and BMI z-score were selected as they are commonly used for assessing overweight and obesity in adolescents. Differences in education systems, modes of delivery of interventions, cultural and contextual differences could affect the relevance and applicability of the findings. Therefore, only studies from high-income countries were included in this review. The definitions for income groups for countries by World Bank (2020) [32] were used to exclude low-and middle-income countries (LMICs). We also included multi-component intervention studies that addressed other issues such as unhealthy diet and physical inactivity if they also reported BMI outcomes. Health education was defined as ‘any combination of learning experiences designed to facilitate voluntary adaptations of behaviour conducive to health’ [33]. By this definition, interventions delivered in an educational setting, which provided information on improving diet and/or physical activity and preventing excess weight gain were included. Educational interventions supplemented by behaviour change techniques and using innovative tools for dissemination such as digital interventions were also included. As schools often have general health education as part of their curriculum, only interventions that were delivered as an addition to existing lessons, with the main component delivered within the school environment, were included. Studies focusing only on specific groups (e.g. adolescents with obesity, or specific medical conditions) were excluded.

**Table 1:** Review inclusion and exclusion criteria

	Criteria	Justification
Inclusion	Observational and experimental studies with a control or comparison group	We aimed to provide a thorough systematic review of recent literature. Though the quality of evidence will be lower for observational studies a detailed and transparent quality assessment of included studies was conducted.
	Participants within the specified age range of 10–19.	The review focuses on adolescents only.
	Studies that report BMI and related outcomes with a pre and post-intervention comparison, baseline to follow up etc.	To identify interventions that bring about a change in outcomes
	High-income countries	Differences in education systems, modes of delivery of interventions, cultural and contextual differences, co-existence of under-and over-nutrition could all affect the relevance and generalisability of the findings. The definitions for income groups for countries by World bank (2020) were used to exclude low and middle-income countries.
	Interventions from year 2006	High quality systematic reviews covered the evidence of school-based interventions up to 2006. We aimed to provide an updated account of interventions with focus on adolescents in school settings.
Exclusion	Studies that do not report change in BMI outcomes	Studies that aim to prevent obesity and overweight or reduce BMI were included. BMI outcomes (BMI, BMI z-score, change in prevalence of overweight and obesity) were selected as the outcomes for this review as an initial scoping search showed that BMI/ BMI Z score were most commonly reported in studies based in schools. BMI is also more feasible for assessment of students in schools.
	Papers published in a language other than English	Translation resources not available
	Specific disease groups targeted (e.g. Diabetes and other endocrine disorders) Interventions targeting only students with overweight and/or obesity at baseline	The review aims to explore interventions with implications for the general population and targeted interventions for different conditions may require different strategies.

### Data extraction and quality assessment

Titles and abstracts were downloaded, and duplicates were removed using EndNote bibliographic software. Three authors (CMJ, PHJ and MB) screened titles and abstracts that met the inclusion criteria. Full texts were then assessed for eligibility by three reviewers (CMJ, PHJ, MB) and any disagreements resolved through discussion with a fourth reviewer (JB). Data were extracted from included studies using a form to capture key information on populations, intervention strategies and results. A modified version of a quality assessment rubric, based on CRD guidance, was used to assess risk of bias in included studies in relation to the review questions. Risk of bias scores ranged from − 6 to + 11 and were categorized into low risk (+ 5 and above), medium risk (+ 1 to + 4) and high risk (0 to − 6). Scores of + 1/ 0/ -1 were given for different criteria (e.g. selection criteria, analytical methods) and tallied to provide a final score for risk of bias. For example, studies were awarded + 1 for randomized controlled trials, 0 for quasi-experimental studies that include a control group, and − 1 for experimental studies that do not use a control group. A full description of the assessment criteria can be found in the supplementary material. The included studies were divided into two sets, and reviewers (CMJ and PHJ) reviewed one set each. Papers identified through an updated search (2018–2020) were reviewed by three reviewers (PHJ, CMJ and MB). To ensure consistency, a third of the papers from each set were cross-checked by another reviewer.

### Statistical analysis

A meta-analysis was conducted for studies presenting data on BMI z-score. Although some issues were present due to heterogeneity of target groups, specific intervention components and how outcome measures were presented, sufficient data were available for meta-analysis in 14 of the 33 included studies [34–47]. Studies not included in the meta-analysis did not provide results for BMI z-score but reported a change in BMI, BMI percentile or prevalence of obesity/ overweight based on calculated BMI or BMI z-score. Heterogeneity was assessed using Cochran’s Q and the percentage of variability due to heterogeneity was quantified using I^2^. To account for the heterogeneity between studies, a random-effects model was used in the meta-analysis. Where follow-up results were recorded at different time points during data extraction, the longest follow-up measure was used, and, where available, sub-group results (based on gender) were obtained. Meta-analysis was conducted on the full dataset from the 14 studies and then repeated according to gender for those studies that reported such findings separately. Due to the limited number of studies eligible for meta-analysis, subset analyses by intervention features, risk of bias and mode of delivery could not be conducted. Funnel plots were created to assess the possibility of publication bias for studies included in the meta-analysis (*n* = 14). All analyses were performed using Stata version 14 (StataCorp LP, College Station, Texas, USA).

## Results

### Results of literature search

**Fig. 1:**
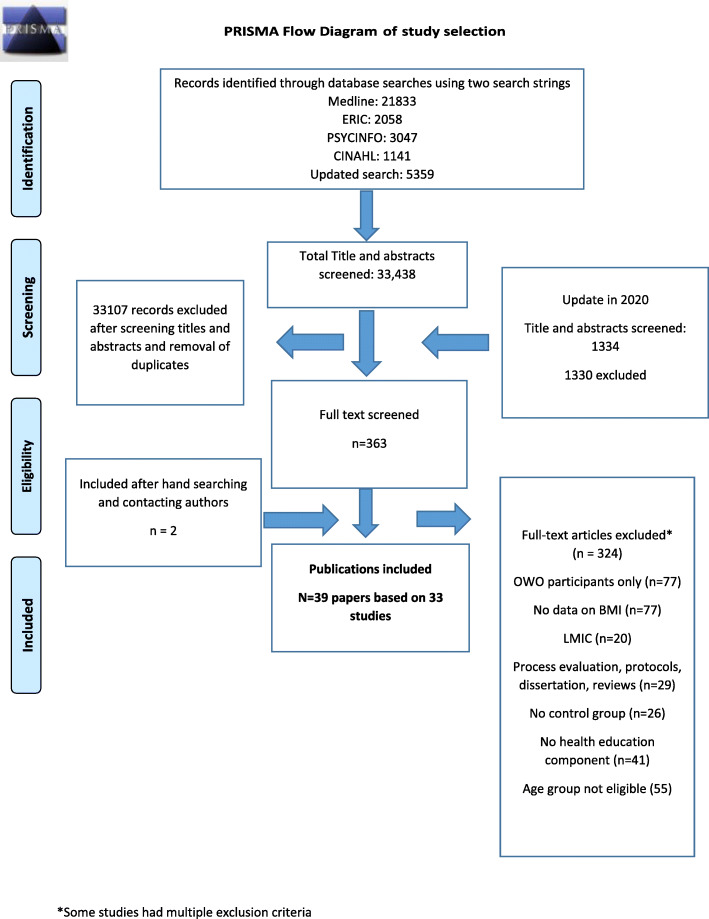
PRISMA Flow Diagram of study selection

The total search retrieved 34,772 records. Following removal of duplicates, 32,828 were screened by title and abstract. The remaining 363 full text articles were screened, of which 39 publications met the inclusion criteria (see Fig. 1 PRISMA flow chart). Some of the publications were based on the same study cohorts but reported different outcomes of the same intervention. Where this occurred, we grouped the publications by study cohort for reporting and analysis.

### Characteristics of included studies

We identified 39 papers, based on 33 studies [34–67]. Six studies recruited adolescent girls only [36, 40, 44, 47, 61, 63] one adolescent boys only [42] and one study included parent-student dyads [66]. Most of the studies (*n* = 27) focused on adolescents aged 10–14 years [34, 36–42, 44–48, 50, 52, 54, 56–66], and six recruited participants from high schools/secondary schools without defining an age range [35, 43, 49, 51, 53, 55]. Eighteen reported the use of behaviour change theory to inform the development of their interventions [36, 38, 40–42, 44, 47, 52–57, 61, 63–66] with the most common theory being social cognitive theory [36, 38, 41, 42, 52, 53, 55, 56, 61].

Most studies evaluated multi-component interventions which, in addition to health education delivered in the classroom, included components such as homework activities, environment modification, physical activity classes, and fruit and vegetable breaks during class (*n* = 26). The remaining seven interventions included health education interventions only [34, 45, 50, 60, 62, 64, 65]. Table 2 provides an overview of the features of included studies and percentage of effectiveness for each component.

**Table 2:** Key characteristics of studies included in the review

Characteristic	Citations	Proportion of Studies N (% of 33)	N and % with Significant BMI Outcomes
**Intervention delivery**			
Teachers	[34–39, 45, 46, 48–50, 52–54, 57, 59, 61, 62]	22 (67%)	12 (54%)
Researchers	[55, 56, 58, 63–65]	6 (18%)	2 (33%)
School Nurse	[44]	1 (3%)	0
School Project Officer	[43]	1 (3%)	1 (100%)
Community based instructor	[47, 66]	2 (6%)	0
Not Stated	[51]	1 (3%)	0
**CPD for teachers Provided**	[34, 36–43, 45, 48–50, 52–54, 57, 59, 61]	19 (58%)	12 (63%)
**Mode of CPD**^a^			
Textbook/Manual	[37, 39, 50, 52, 54, 57, 59]	7 (21%)	3 (43%)
Seminars/Workshop	[36, 38, 40–43, 53, 54, 61]	9 (27%)	6 (67%)
Training on Intervention Delivery only	[34, 38, 39, 45, 52]	5 (15%)	3 (60%)
Not stated	[48, 49]	2 (6%)	1 (50%)
**Included Parental Involvement**	[36, 37, 39, 40, 42, 43, 46, 48, 49, 52–56, 63, 66]	16 (48%)	9 (56%)
**Mode of Parental Involvement**			
Text messages/ Emails	[37, 46]	2 (6%)	1 (50%)
Newsletters/Information leaflets	[36, 40, 42, 43, 49, 52, 53, 56]	8 (24%)	5 (63%)
Family workshops/ Parent Engagement	[39, 48, 54, 55, 63, 66]	6 (18%)	2 (33%)
**Engagement components**^b^			
App/Website/CD-ROM/ text messages/ social media	[35, 36, 40, 42, 43, 46, 50, 55, 59, 63–66]	15 (45%)	5 (83%)
Computer-Tailored Advice	[40, 47, 50, 57, 59]	5 (15%)	1 (20%)
After-school/out of class	[35, 36, 38, 40–44, 46, 48, 55, 59, 63]	13 (39%)	5 (38%)
Games	[34, 45]	2 (6%)	1 (50%)
**Other Intervention Features**			
Environmental modification/ School Policy Change	[37, 39, 40, 42, 46, 48, 49, 52, 59, 61]	10 (30%)	6 (60%)
Community Programme	[43, 55]	2 (6%)	1 (50%)
Exercise/ PA classes	[36, 42–44, 47, 51–53, 56, 58, 63, 66]	12 (36%)	4 (33%)
**Study Design**			
RCT	[34–36, 38–42, 45–48, 50, 52–59, 62, 63]	23 (70%)	9 (39%)
Non-RCT	[37, 41, 43, 49, 51, 60, 61, 64–66]	10 (30%)	5 (50%)
**Intervention Duration**			
< 6 months	[34, 38, 41, 42, 45, 47, 48, 50, 53–56, 58, 60, 62, 65, 66]	17 (52%)	8 (47%)
6 months- 1 year	[36, 44, 51, 59, 63]	5 (15%)	0
1–2 years	[35, 37, 39, 40, 49, 57, 61, 64]	8 (24%)	4 (50%)
> 2 years	[43, 46, 52]	3 (9%)	2 (67%)
**Intervention type**			
Health education only	[34, 45, 50, 60, 62, 64, 65]	7 (21%)	2 (29%)
Multi-component	[35–45, 46–49, 51–59, 61, 63, 66]	26 (79%) [67]	12 (48%)

^a^Training on intervention delivery was provided for all interventions. Additional training was also provided for some studies, indicated in the table

^b^Some studies may include more than one facilitator, delivery mode or intervention strategy

Thirteen of the studies included had an after-school component such as monitoring diet and physical activity habits at home, providing additional resources to support behaviour change at home (e.g., recipe cards), community activities and social events [35, 36, 38, 40, 43, 46–48, 55, 57, 59, 63, 65, 66]. Twelve studies provided additional sessions for organised sports or clubs to increase exercise or physical activity [36, 42–44, 47, 51–53, 56, 58, 63, 64]. Table 3 describes the characteristics of each intervention. Quality assessment scores are reported in Table 4. A quality assessment of the 33 studies included in this review indicated that 14 had a low risk of bias compared with 10 with medium and nine with a high risk of bias. Risk of Bias scores ranged from − 3 [49, 61] to + 11 [47]. As most studies were multi-component, developing a narrative summary with exclusive groups based on characteristics was not feasible. For discussion, we present groups based on their dominant intervention components.

**Table 3:** Summary of findings from included studies

Author, Year, Country, Name of Cohort/ study, Citation	Study design, Participant characteristics^a^	Key Intervention characteristics	Main findings and results for BMI/BMI z-score outcomes ^b^
**Low Risk of Bias**			
Robbins, 2020 [66], USA GOAL	Pre test–post-test quasi-experimental design Mean age 11.6 (0.09) Intervention: *n* = 38 Control: *n* = 43 A total of 81 parent-student dyads were recruited	• Primary outcomes: feasibility and acceptability (qualitative), MVPA, diet quality, psychosocial variables (motivation, self-efficacy, and perceived social support for PA and healthy eating • Duration of intervention: 12 weeks • Follow up post intervention • Teacher training: not needed as delivered by instructors and managers with experience in nutrition programmes (for the after-school clubs) • Parental involvement: parent- student dyad combined meetings • Digital component: Facebook participation with weekly healthy eating and PA habit forming tasks for parents to help adolescents with MVPA and diet • Behaviour change theory: Self-determination theory and information-motivation-behaviour skills (IMB) model	Proportion of overweight and obese: Intervention group: Baseline and post intervention 55.9% (*p* = 1.00) Control group Baseline 40.5% Follow up 50.0% (*p* = 0.13)
Robbins 2020 [67], USA *and* Pfeiffer et al. (2019) [47], USA Girls on the Move	Group RCT Mean age 12.07 (girls) Intervention: *n* = 593 Control: *n* = 601 (schools in low-income areas)	• Primary outcome: MVPA, BMI Z score, Percentage body fat and cardiorespiratory fitness • Duration of intervention: 17 weeks • Follow up: immediate post-intervention and 1–4 weeks after intervention was completed • Delivered by trained nurse and community-based instructors • Digital component: one interactive internet-based session providing motivational and feedback messages • Parental involvement: None during intervention period • Behaviour Change Theory: Health promotion model and Self-determination theory	No significant differences occurred for BMI z score post intervention (B = − 0.02, *P* = 0.232) Intervention Baseline 1.30 (0.74) Post intervention 1.30 (0.74) Control Baseline 1.42 (0.73) Follow up 1.44 (0.73) Unadjusted effect Size Cohen d = − 0.03 (subset analysis based on attendance (days/ week) provided but did not show any effect on BMI outcomes. 1–4 week Follow up results: Control (766): 12.05 years. Intervention (753): 12.05 years. No significant between-group differences in BMI-z existed at post intervention (B = − 0.02, .191, 95% confidence interval [CI; − 0.05-0.01]).
Wadolawska, 2019 [60], Poland	Non-randomised study with control group Mean age: 11.9 (11.9–12.0) Intervention *n* = 319 Control *n* = 145	• Primary outcome: Physical activity, sedentary time, diet and body composition (z-Waist to height Ratio, Z BMI for age, Waist circumference) • Duration of intervention: 3 weeks • Follow up: 9 months after baseline • Delivered by researchers • Digital component: None • Parental involvement: None • Behaviour change theory: Integrated theory	Change in z-BMI-for-age SDs (Follow up – baseline): Int: − 0.01 (− 0.07 to 0.04) Con: 0.03 (− 0.01 to 0.07) Difference: − 0.04
Bogart, 2016^d^ [48] USA	RCT Mean age: 12.2 (0.68) Intervention: *n* = 829 Control: *n* = 539	• Primary outcome: BMI percentile • Duration of intervention: 5 weeks • Follow up after intervention: 2 years • Teacher training: Delivered by peer leaders and teachers (training given to peer leaders). • Parental involvement: Homework activities with parents • Behaviour change theory: Social cognitive theory and community based participatory research	BMI percentile (intervention versus control): adjusted difference = − 0.98 (SE 1.01), *p* = 0.33 Subset analysis (students obese at baseline) BMI percentile adjusted b = −2.33 (SE 0.83; *P* = 0.005) compared with control students.
Lubans, 2016 [42] and Smith 2014 [68], Australia ATLAS	Cluster RCT Mean age: 12.7 (0.5) Intervention: *n* = 121 Control: *n* = 143	• Primary outcome: BMI, waist circumference • Duration of intervention: 8 months • Follow up: immediately after intervention and 18 months from baseline • Teacher training: two 6-h workshops • Digital component: smartphone app and website. • Parental involvement: Parent newsletters. • Behaviour change theory: self-determination theory and social cognitive theory	BMI z-score (adjusted mean difference) at 18 months from baseline: 0.04 (95%CI: − 0.07 to 0.14)
Melnyk, 2015 [53], USA^c^ COPE	Cluster RCT Mean age: 14.74 (0.73) Intervention: *n* = 358 Control: *n* = 421	• Primary outcome: BMI and depressive symptoms • Intervention duration: 15 weeks • Follow up after intervention: 12 months • Teacher training: full day training workshop • Digital component: None reported • Parental involvement: Newsletter provided and students were expected to discuss with parents • Behaviour change theory: cognitive theory	BMI at 12 months adjusted mean COPE teens (24.94, SE 0.12) Control group (25.48, SE 0.11) *p*-value for difference = 0.001
Viggiano, 2015 [45], Italy^c^ Kaledo	RCT Mean age 13.0 (12.9–13.0) Intervention: *n* = 1663 Control: *n* = 1447	• Primary outcomes: dietary behaviours and BMI z-scores • Duration of intervention: 20 weeks • Follow up: 6 and 18 months post baseline • Teacher training: teachers trained in playing the game • Digital component: None reported • Parental involvement: None reported • Behaviour change theory: Not specified	BMI z-scores At 6 month follow-up: Middle schools: Intervention: 0.49 (95%CI: 0.45 to 0.53) Control: 0.58 (95%CI: 0.54 to 0.62) p-value for difference = 0.007 High schools: Intervention: 0.35 (95%CI: 0.29 to 0.40) Control: 0.57 (95%CI: 0.51 to 0.63) p-value for difference < 0.001 At 18 month FU: Middle schools: Intervention: 0.40 (95%CI: 0.28 to 0.52) Control: 0.57 (95%CI: 0.44 to 0.71) p-value for difference = 0.057 High schools: Intervention: 0.13 (95%CI: − 0.09 to 0.34) Control: 0.61 (95%CI 0.31 to 0.90) p-value for difference = 0.015
Dewar, 2013 [36], Australia Neat Girls	RCT Mean age 13.2 (0.5) Intervention *n* = 178, Control *n* = 179 Girls only	• Primary outcomes: BMI • Duration of intervention: one year • Follow up at 12 months and 24 months (12 months post intervention) • Teacher training: 1-day training workshop. • Digital component: SMS • Parental involvement: Parent newsletters • Behaviour change theory: Social cognitive theory	Adjusted BMI z-score change at 12 month post intervention: − 0.12 (95%CI: − 0.27, 0.04)
Ezendam, 2012 [50], Netherlands FATaintPHAT	RCT Mean age (control group): 12.7 (0.7 Control: 340 Intervention: 395	• Primary outcomes: Waist circumference, BMI and fitness • Duration of intervention: 10 weeks • Follow up at 4 months and 2 years • Teacher training: manual provided • Digital component: primarily an internet-based intervention • Parental involvement: None reported • Behaviour change theory: Theory of Planned Behaviour	Not reported for 4-month follow-up. BMI change at 2 years between group difference: − 0.14 (95%CI: − 0.17 to 0.45)
Robbins, 2012 [44], USA Pilot study *Girls on the Move*	Quasi-experimental study Mean age: 11.4 years Intervention: *n* = 37 Control: *n* = 32	• Primary outcomes: BMI, physical activity, and cardiovascular fitness • Duration of intervention: 6 months • Follow up after intervention: immediately post intervention • Teacher training: Motivational Interviewing training for the school nurse • Digital component: None reported • Parental involvement: None reported • Behaviour change theory: health promotion model	BMI z-score change: Intervention: 0.06 (0.18) Control: 0.12 (0.18) Adjusted difference: − 0.04 (*p* = 0.24)
Prins, 2012 [57], Netherlands	RCT Mean age: 12.7 (0.5) Intervention: 281 Control: 254	• Primary outcomes: Compliance with MVPA guideline and minutes spent in MVPA • Duration of the intervention: Not reported • Follow up at one and six months post intervention • Teacher training: Manual provided • Digital component: Computer tailored PA promotion • Parental involvement: None reported • Behaviour change theory: Socio-ecological model	Unstandardized regression coefficient (95% CI) for prevalence of overweight and obesity at 6 month follow-up: 0.16 (95%CI: − 1.01 to 1.13).
The HEALTHY study group, 2010 [52], USA^c^	RCT Mean age 11.3 (0.6) Intervention: 4603 Control: 2296	• Primary outcomes: Risk of diabetes (BMI, waist circumference, fasting glucose and insulin levels), combined prevalence of OWO • Duration of intervention: 3 years • Follow up at 3 years from baseline • Teacher training: 4 h training with companion manual • Digital component: None • Parental involvement: newsletters matching theme of semester • Behaviour change theory: Social cognitive theory	Change in BMI z-score: Intervention: − 0.05 Control group: − 0.01
Peralta, 2009 [56], Australia FILA study (Fitness Improvement Lifestyle Awareness)	RCT Mean age 12.5 (0.4) Intervention: *n =* 16 Control: *n =* 17 Boys only	• Primary outcome: BMI • Follow up after intervention: 6 months • Teacher training: None (delivered by researcher) • Digital component: None reported • Parental involvement: Newsletters • Behaviour change theory: social cognitive theory	Adjusted BMI difference between intervention and control: − 0.2 (95%CI: − 0.78 to 0.39), *p* = 0.5
Singh, 2007 [59], The Netherlands DOiT (Dutch Obesity Intervention in Teenagers)	RCT Mean age (control) 12.8 (0.51) Total *n =* 978	• Primary outcomes: BMI, measures of body fatness and aerobic fitness • Duration of intervention: 8 months • Follow up immediately after intervention • Teacher training: Teachers received a manual to support them in delivering the intervention • Digital component: Individual advice provided by CD rom • Parental involvement: None reported • Behaviour change theory: Not specified	BMI (difference between intervention and control in change between groups): Girls: − 0.05 (95%CI: − 0.18 to 0.08) Boys: − 0.02 (95%CI: − 0.11 to 0.16)
**Medium Risk of Bias**			
Ermetici, 2016 [37], Italy^c^ EAT Study	Non-randomised quasi-experimental study Mean age 12.5 (0.4) Intervention: *n* = 262 Control: *n* = 225	• Primary outcome: BMI Z-score • Intervention duration: 2 school years • Follow up after intervention: Immediate post intervention • Teacher training: Text book to aid lessons • Digital component: Automated text messages • Parental involvement: Text messages • Behaviour change theory: not specified	After 2 years, BMI z-score (adjusted difference): - 0.18 (95%CI: − 0.27 to − 0.09), *p* = 0.003
Wilksh, 2015, Australia [62]	Four arm RCT with multiple educational modules Mean age: 13.21 (0.68) Media Smart (MS): *N* = 269 Life Smart (LS): *N* = 347 HELPP (HP): *N* = 225 Control (C): *N* = 473	• Primary outcome: risk of eating disorders • Duration of intervention (LifeSmart): 5 weeks • Follow up after intervention: post program, 6 month and 12 months • Teacher training: None reported • Digital component: None reported • Parental involvement: None reported • Behaviour change theory: not specified	Group by time effect showed no significant effects on BMI for boys or girls at 12 month follow up.
Lazorick, 2015 [41], US^c^ The MATCH Intervention	Two armed quasi-experimental study Mean age: 13.3 (0.79) Intervention: *n* = 189 Control: *n* = 173	• Primary outcome: BMI and BMI Z-score • Intervention duration: 14 weeks • Follow up after intervention: post intervention and one year • Teacher training: One day of teacher training provided and a two-day orientation • Digital component: None reported • Parental involvement: None reported • Behaviour change theory: Social cognitive and self-determination theory	Mean change BMI z-score immediately post intervention Intervention: − 0.06 (95%CI: − 0.08 to − 0.03) Control: 0.02 (95%CI: − 0.004 to 0.05) *p*-value for difference < 0.001
Grydeland, 2014 [40], Norway^d^ HEIA Intervention Study	Two armed RCT. Mean age = 11.2 (0.3) Intervention: *n* = 465 Control: *n* = 859	• Primary outcome: BMI and BMI Z-score • Intervention duration: 20 months • Follow up after intervention: immediate post intervention • Teacher training: PE teachers were enrolled in a course • Digital component: Computer tailored individual advice • Parental involvement: Parent-based fact sheets • Behaviour change theory: socio-ecological framework	Post intervention BMI z-score (adjusted for baseline values) Girls Intervention: − 0.8 (95%CI: − 0.14, − 0.02) Control: 0.03 (95%CI: − 0.01, 0.08) p-value for difference = 0.003 Boys Intervention: − 0.01 (− 0.07, 0.05) Control: − 0.05 (95%CI: − 0.09, − 0.00) p-value for difference = 0.32 Total Intervention: − 0.04 (95%CI: − 0.09, 0.00) Control: − 0.01 (95%CI: − 0.04, 0.02) p-value for difference = 0.227
Bonsergent, 2013 [35], France PRALIMAP	RCT Mean age 15.6 (0.7) Intervention: 3424 Control: 2947	• Primary outcomes: BMI and BMI Z-score • Duration of intervention: 24 months • Follow up: at 12 months and immediately post intervention • Teacher training: None reported • Digital component: None reported • Parental involvement: None reported • Behaviour change theory: None reported	BMI z-score change at 12 months post intervention: β = 0.004 (95%CI: − 0.026, 0.034)
Fairclough, 2013 [38], UK^c^ The CHANGE! Intervention	RCT Mean age (control group): 10.7 (0.3) Control: 117 Intervention: 89	• Primary outcomes: Waist circumference, BMI and BMI z-score • Duration of intervention: 20 weeks • Follow up at immediately post intervention (20 weeks) and 30 weeks • Teacher training: 4 h in how to deliver the curriculum • Digital component: CD ROM • Parental involvement: None reported • Behaviour change theory: Social cognitive theory	BMI z-score adjusted change at 20 weeks: β = − 0.04 (95% CI: − 0.22, 0.15), *p* = 0.68 BMI z-score adjusted change at 30 weeks: β = − 0.24 (95% CI: − 0.48, − 0.003), *p* = 0.04
Williamson, 2012 [46], USA	RCT Mean age: 12.9 (1.2) Intervention: PP = 511; PP+ SP = 516 Control: 307	• Primary outcomes: Percentage body fat and BMI z-score • Duration of intervention: 28 months. • Follow up at 18 months and 28 months (immediately post intervention). • Teacher training: None reported • Digital component: Online platform • Parental involvement: Emails to parents. • Behaviour change theory: Not reported.	Adjusted difference between control and intervention BMI z-scores at 28 months: Boys: β = − 0.034 Girls: β = − 0.035
Neumark-Sztainer, 2010 [55], US * New Moves*	RCT Mean age: 15.8 (1.2) Intervention: 182 Control: 174 Girls only	• Primary outcomes: Physical activity levels • Duration of intervention: one school year. • Follow-up at immediately post intervention and 9 months post intervention. • Teacher training: None reported. • Digital component: None reported. • Parental involvement: Parent outreach and parent-daughter retreat days. • Behaviour change theory: Social cognitive theory.	Adjusted BMI difference between groups at 9 month post intervention follow-up: − 0.10, *p* = 0.446
Mihas, 2009 [54], Greece^c^ VYRONAS study	RCT Mean age (control) 13.3 (0.9) Intervention: *n* = 98 Control: *n* = 93	• Primary outcomes: dietary habits and BMI • Duration of intervention: 12 weeks • Follow ups after intervention: 15 days and 12 months • Teacher training: materials and two 3-h seminars • Digital component: none reported • Parental involvement: nutrition education and behaviour change • Behaviour change theory: social learning theory	No change in BMI at 15 days Mean BMI (adjusted) at 12 months vs baseline: Int: 23.3 (SD 2.8) vs 24.0 (SD 3.1) *p* < 0.001 Con: 24.8 (SD 3.8) vs 24.3 (SD 3.3) *p* = 0.36
Young, 2006 [63], USA	RCT Mean age 13.8 (0.5) Intervention: *n* = 111 Control: *n* = 99 Girls only	• Primary outcomes: physical activity and markers of cardiovascular disease risk factors • Duration of intervention: 8 months • Follow up immediately after intervention • Teacher training: not needed as intervention delivered by research staff • Digital component: none reported • Parental involvement: family workshops, monthly newsletters and parent/child homework • Behaviour change theory: social action theory	Adjusted mean BMI change: Intervention: 0.3 (SE 0.2) *p =* 0.2 Control: 0.2 (SE 0.2) *p* = 0.34 Between group *p* = 0.81
**High risk of Bias**			
Benitez-Andrades (2020),^d^ [65] Spain	Non-randomised pre-post quasi experimental study design with control group Mean age: 12.8 for C and 12.6 for I Intervention: *n* = 139 Control: *n* = 91	• Primary outcome: BMI age-adjusted percentile, physical activity, eating habits • Duration of intervention: 14 weeks • Follow up: immediately after intervention only • Delivered by researchers • Digital component: Facebook-based intervention • Parental involvement: None • Behaviour Change Theory: Not specified	Intervention group: BMI age-adjusted percentile (≥50 initial i.e. overweight) Mean Pre: 77.59 Mean post: 72.85 Z: − 5.394 *p* = 0.000 Control group: BMI age-adjusted percentile (≥50 initial) Mean pre: 78.09 Mean post: 77.49 Z = 0.241 *p* = 0.809
Froberg, 2018 [64], Sweden	Non-randomised study with control group Mean age: 12.8 (0.5) Intervention: 51 Control: 47	• Primary outcome: physical activity, food habits, and behaviour change • Duration of intervention: 2 years • Follow up: 2 years and 4 months from after baseline • Delivered by researchers • Digital component: Facebook group for communication between researchers and students, however main intervention was delivered in class. • Parental involvement: None • Behaviour change theory: empowerment-based health promotion, shared decision making	Non-adjusted mean difference in BMI between intervention and control: 1.9 (95% CI: 0.035, 3.76). Change in prevalence for overweight: − 0.8% Change in prevalence for obesity: 1.3%
Busch, 2015 [49], The Netherlands^d^ Utrecht Healthy School (UHS)	Non-randomised controlled trial Age group: high school students 1 year (*N* = 969) 2 years (*N* = 605).^a^	• Primary outcome: BMI, health behaviour and psychosocial health • Intervention duration: The UHS was integrated into the school curriculum for 2 years • Follow up after intervention: 1 and 2 years from baseline • Teacher training: For teachers and head teachers • Digital component: None reported • Parental involvement: participation in school projects • Behaviour change theory: not specified	Adjusted BMI change from baseline for interventions schools compared with control schools School A Year 1: β = − 0.48 (*p* < 0.05) Year 2: β = − 0.58 (*p <* 0.05) School B Year 1: β = − 0.05 (*p* > 0.05) Year 2: β = − 0.43 (*p >* 0.05)
Millar, 2011 [43], USA ^c^	Quasi-experimental study Mean age: 14.6 (1.42) Intervention: *n* = 1276 Control: 778	• Primary outcomes: BMI, BMI z-score and body composition • Duration of intervention: Not reported • Follow up at (m; SD) 2.3 (0.68) years from baseline. • Teacher training: CPD for PE teachers. • Digital component: None reported. • Parental involvement: parent information, family and home environment • Behaviour change theory: Not reported.	Adjusted difference between intervention and control BMI z-scores at follow-up: − 0.07 (SE 0.03), *p* = 0.03
Graham, 2008 [51] Schneider, 2007 [69] USA	Non-randomised controlled trial Mean age 15.04 (0.79) Intervention: *n* = 63 Control: *n* = 59 Girls only	• Primary outcomes: Cardiovascular fitness and physical activity levels • Duration of intervention: 9 months • Follow up after immediately after intervention • Teacher training: No teacher training described, unclear who delivered the intervention • Digital component: None reported • Parental involvement: None reported • Behaviour change theory: not specified	No significant difference (*p* = 0.1) between groups in change in BMI percentile
Webber, 2008 [61], US Trial of Activity for Adolescent Girls (TAAG)	Cluster RCT Mean ages: 6th graders 12.0 8th graders 14.0 *n* = 1721 (6th grade 2003) *n* = 3504 (8th grade 2005) *n* = 3502 (8th grade 2006) Girls only	• Primary outcomes: physical activity levels and body composition measurements • Duration of intervention: 2–3 years • Follow-ups: 2 and 3 years post baseline • Teacher training: PE teachers and Program Champions were trained by TAAG investigators • Digital component: None reported • Parental involvement: None reported • Behaviour change theories: operant learning theory, social cognitive theory, organizational change theory, and diffusion of innovation model	BMI mean difference: 6th grade (2003): − 0.2 (95%CI: − 1.0 to 0.6) 8th grade (2005): − 0.2 (95%CI: − 0.6 to 0.2) 8th grade (2006): 0.1 (95%CI: − 0.4 to 0.7)
Foster, 2008 [39], US^c^(SNIP study (School Nutrition Policy Initiative)	RCT Mean age (control) 11.2 (1.0) Intervention: *n* = 749 Control: *n* = 600	• Primary outcomes: incidence of overweight and obesity • Duration of intervention: 2 years • Follow-up immediately after intervention • Teacher training: Teachers were offered up to 10 h of training per year • Digital component: None reported • Parental involvement: Parent outreach • Behaviour change theory: Not specified	BMI (adjusted difference): − 0.04 (95%CI: − 0.27 to 0.19), *p* = 0.71 BMI z-score (adjusted difference): − 0.01 (95%CI: − 0.08 to 0.06), *p* = 0.80 Predicted odds ratio for incidence of overweight (adjusted): 0.67 (95%CI: 0.47 to 0.96) *p <* 0.05
Rosenbaum, 2007 [58], USA ^c^	RCT Mean age (control) 13.6 (0.2) Intervention: *n* = 49 Control: *n* = 24	• Primary outcomes: Markers of insulin sensitivity and inflammation • Duration of intervention: 3–4 months • Follow up immediately after intervention • Teacher training: None (delivered by researchers) • Digital component: None reported • Parental involvement: None reported Behaviour change theory: Not specified	BMI at baseline and follow-up: Control: 24.3 (SD: 1.8) to 24.8 (SD: 1.9) *p* ≥ 0.05 Intervention: 24.7 (SD: 1.4) to 24.0 (SD:1.5) *p <* 0.05
Amaro, 2006 [34], Italy, Kaledo pilot (See Viggiano, 2015 [45])	RCT Mean age (control) 12.5 (0.7) Intervention: *n* = 153 Control: *n* = 88	• Primary outcomes: dietary behaviours and BMI Z-score • Duration of intervention: 24 weeks • Follow up: immediately after intervention • Teacher training: teachers trained in playing the game. • Digital component: None reported • Parental involvement: None reported • Behaviour change theory: Not specified	BMI z-score (adjusted mean controlling for baseline values): Intervention: 0.35 (95CI%: 0.30 to 0.39) Control: 0.41 (95%CI: 0.35 to 0.47)

^a^**Some studies only reported age by group or group and sex, but were similar in both groups; Where sample size for intervention and control groups are not reported, total size is presented**

^b^**MD (95%CI),** ***p*-value reported when available**

^c^Studies with significant effects on BMI outcomes

^d^Significant effect in a subset analysis

**Table 4:** Quality assessment of included studies

Study (Author, year)	Study Design	Randomisation	Blinding assessors	Blinding participants	Baseline	Selection criteria	Approach to Selection	Loss to follow up	Assessment of BMI, BMI Z score	Intervention delivery	Point estimates and measures of variability primary outcome	ITT^a^ analysis	Analytical methods	Confounding	Sample size	Total score^b^	Risk of Bias
Amaro (2006) [34]	+ 1	0	− 1	0	0	0	0	0	+ 1	0	0	−1	+ 1	0	−1	0	High
Benitez-Andrades (2020) [65]	0	0	0	0	+ 1	0	0	−1	0	+ 1	− 1	− 1	+ 1	+ 1	− 1	0	High
Bogart (2016) [48]	+ 1	+ 1	0	0	0	+ 1	0	0	0	0	0	+ 1	+ 1	+ 1	+ 1	+ 7	Low
Bonsergent (2013) [35]	+ 1	−1	−1	0	0	+ 1	0	−1	+ 1	− 1	+ 1	− 1	+ 1	+ 1	0	+ 1	Medium
Busch (2015) [49]	+ 1	−1	−1	0	−1	0	0	0	+ 1	−1	+ 1	− 1	+ 1	0	−1	−3	High
Dewar (2013) [36]	+ 1	0	+ 1	+ 1	+ 1	+ 1	0	−1	+ 1	− 1	+ 1	+ 1	+ 1	+ 1	+ 1	+ 9	Low
Ermetici (2016) [37]	0	−1	− 1	0	+ 1	+ 1	0	+ 1	+ 1	+ 1	+ 1	−1	+ 1	+ 1	−1	+ 4	Medium
Ezendam (2012) [50]	+ 1	+ 1	0	0	0	+ 1	0	0	+ 1	+ 1	+ 1	+ 1	+ 1	+ 1	−1	+ 8	Low
Fairclough (2013) [38]	+ 1	+ 1	−1	− 1	0	+ 1	0	0	+ 1	+ 1	−1	−1	+ 1	+ 1	− 1	+ 2	Medium
Foster (2008) [39]	+ 1	−1	− 1	0	0	0	0	−1	0	−1	+ 1	− 1	+ 1	+ 1	0	− 1	High
Froberg (2018) [64]	0	−1	− 1	0	− 1	+ 1	0	0	+ 1	0	−1	− 1	− 1	−1	− 1	−6	High
Graham (2008) [51]	0	−1	−1	− 1	0	+ 1	− 1	+ 1	+ 1	0	+ 1	− 1	+ 1	− 1	− 1	−2	High
Grydeland (2014) [40]	+ 1	− 1	− 1	0	+ 1	+ 1	0	+ 1	+ 1	− 1	+ 1	−1	+ 1	0	+ 1	+ 4	Medium
HEALTHY study (2010) [52]	+ 1	+ 1	+ 1	0	+ 1	+ 1	0	0	+ 1	+ 1	+ 1	−1	+ 1	−1	+ 1	+ 8	Low
Lazorick (2015) [41]	0	−1	− 1	0	0	+ 1	0	+ 1	+ 1	−1	+ 1	−1	+ 1	+ 1	− 1	+ 1	Medium
Lubans (2016) [42]	+ 1	+ 1	0	+ 1	0	+ 1	0	0	+ 1	−1	+ 1	+ 1	+ 1	0	+ 1	+ 8	Low
Melnyk (2015) [53]	+ 1	0	+ 1	+ 1	−1	+ 1	0	0	+ 1	−1	+ 1	+ 1	+ 1	+ 1	+ 1	+ 8	Low
Mihas (2009) [54]	+ 1	+ 1	−1	0	+ 1	+ 1	0	+ 1	0	−1	+ 1	−1	+ 1	+ 1	+ 1	+ 3	Medium
Millar (2011) [43]	0	−1	− 1	0	0	0	0	−1	+ 1	− 1	+ 1	− 1	+ 1	+ 1	0	− 1	High
Neumark-Sztainer (2010) [55]	+ 1	−1	− 1	0	0	+ 1	0	+ 1	+ 1	−1	+ 1	−1	+ 1	+ 1	− 1	+ 2	Medium
Peralta (2009) [56]	+ 1	+ 1	+ 1	−1	−1	+ 1	0	+ 1	+ 1	0	+ 1	+ 1	+ 1	−1	−1	+ 5	Low
Prins (2012) [57]	+ 1	+ 1	0	0	0	0	0	0	+ 1	−1	+ 1	+ 1	+ 1	− 1	+ 1	+ 5	Low
Pfeiffer 2019 [47]	+ 1	0	0	0	+ 1	+ 1	+ 1	+ 1	+ 1	0	+ 1	+ 1	+ 1	+ 1	+ 1	+ 11	Low
Robbins (2012) [44]	+ 1	−1	−1	0	+ 1	+ 1	0	+ 1	+ 1	0	+ 1	+ 1	+ 1	0	+ 1	+ 7	Low
Robbins 2020 [66, 67] GOAL	0	0	−1	0	0	+ 1	0	+ 1	+ 1	+ 1	+ 1	+ 1	+ 1	+ 1	−1	+ 6	Low
Rosenbaum (2007) [58]	+ 1	−1	− 1	0	+ 1	− 1	0	0	+ 1	0	+ 1	−1	+ 1	−1	− 1	−1	High
Singh (2007) [59]	+ 1	−1	− 1	0	0	+ 1	0	0	+ 1	0	+ 1	+ 1	+ 1	0	+ 1	+ 5	Low
Viggiano (2015) [45]	+ 1	+ 1	−1	+ 1	+ 1	0	0	−1	+ 1	+ 1	0	−1	+ 1	0	+ 1	+ 5	Low
Wadolowska (2019) [60]	0	−1	0	0	+ 1	+ 1	0	0	+ 1	+ 1	+ 1	−1	+ 1	+ 1	+ 1	+ 6	Low
Webber (2008) [61]	+ 1	−1	− 1	0	− 1	+ 1	0	0	−1	− 1	0	− 1	+ 1	− 1	+ 1	−3	High
Wilksh (2015) [62]	+ 1	0	−1	0	0	+ 1	0	0	+ 1	0	+ 1	−1	+ 1	0	0	+ 3	Medium
Williamson (2012) [46]	+ 1	−1	−1	0	0	+ 1	0	−1	+ 1	0	+ 1	+ 1	+ 1	−1	+ 1	+ 3	Medium
Young (2006) [63]	+ 1	−1	− 1	0	− 1	+ 1	0	+ 1	+ 1	0	+ 1	−1	+ 1	− 1	0	+ 1	Medium

^a^*ITT* Intention to treat

^b^Details on scoring rubric in supplementary material

#### Mode of intervention delivery

Most interventions were delivered by teachers (*n* = 22) [34–42, 45, 46, 48–50, 52–54, 57, 59, 61, 62] followed by researchers [35, 55, 56, 58, 60, 63–65], school nurses [44, 47] and in others a school project officer and physical education teacher [43] or project managers [66]. The study by Bogart et al. trained ‘peer leaders’, in addition to teachers, to promote and model healthy behaviours and engage other students [48, 70]. Nineteen studies reported training the teachers to deliver the intervention through a variety of means including workbooks or other training materials and face-to-face sessions [34, 36–43, 45, 48–50, 52–54, 57, 59, 61]. For example, the Health in Adolescents (HEIA) Intervention [40] included two courses in physical education based on a previously validated programme for teachers [71]. Millar et al. provided continuing professional development (CPD) programmes for physical education teachers [43]. CPD was defined as the skills, knowledge and experience gained by teachers beyond any initial formal or informal training.

#### Parental involvement

Sixteen interventions had parental involvement [36, 37, 39, 40, 42, 43, 46, 48, 49, 52–56, 63, 66]. The modes of parental involvement are summarized in Tables 2 and 3. Busch et al. conducted an educational intervention integrated into the regular curriculum [49]. The study by Mihas et al. aimed to improve knowledge, behavioural capability, expectations and self-efficacy [54]. Parents were also encouraged to improve their own dietary behaviours. Grydeland et al. collaborated with school principals, teachers, school health services and parent committees [40]. Teachers delivered the lessons and handed out monthly factsheets to parents and an activity box to students with sports equipment and toys to promote physical activity. Parents in the study conducted by Bogart et al.*,* also engaged in homework activities with the students, which included completing worksheets on food preferences among different members of the family and types of fruits and vegetables kept at home [48]. Finally, three of the studies only provided information to parents [37, 43, 53]. Parental involvement was a key component of the Guys/ Girls Opt for Activities for Life (GOAL) intervention [66] which included parent-adolescent dyads for group meetings targeting self-efficacy, social support and motivation. The meetings aimed to assist parents in supporting students’ physical activity and healthy eating habits through discussion on behavioural strategies as well as healthy cooking sessions.

#### Digital interventions

Sixteen studies used digital media such as apps, websites, CD-ROMs and computer-tailored information [35–38, 40, 42, 43, 46, 50, 55, 59, 63–66]. The HEIA intervention included the provision of information to increase awareness of recommended physical activity levels and fruit and vegetable consumption [40]. They also provided tailored advice to students (a subgroup within the intervention) on how to change dietary habits, screen time and physical activity levels. An intervention in Italian schools included 16 health-promoting lessons (delivered by nutritionists) along with text messages for daily exercise and diet advice, as well as environmental modification (e.g. vending machines for healthy food items) [37]. This quasi-experimental study evaluated an intervention in which automated text messages were sent to students and parents three times a week, close to mealtimes, to promote discussions in the family related to healthy eating habits. The FATaintPHAT intervention consisted of a computer-tailored intervention to help prevent excessive weight gain by improving diet, reducing sedentary behaviours and increasing physical activity, with additional modules on weight management [50]. The modules also included specific goal-setting and action planning with normative and comparative (with peers) feedback. Three studies also used social media (such as Facebook) to provide a platform for communication with the researcher (who delivered the intervention) [64], information on sessions [64], and weekly Facebook participation for parents in the GOAL programme [66]. Another intervention [65] used a social-network based eHealth intervention to improve diet and physical activity habits. The participants could use the social network platform to develop friendships and interact with each other while receiving information about nutrition and physical activity. They were also given rewards for improving their habits.

#### Change in environment

Ten studies included measures to improve the environment, in addition to educational components, or school policy change to increase accessibility and/or improve facilities in schools for sports, social marketing and providing healthier meals [37, 39–41, 43, 49, 52, 53, 58, 70]. Three studies also applied environmental modification in neighborhoods through community activities or providing information on improving home/neighborhood environment [35, 49, 59].

Millar et al. provided a school-based intervention with a community component focusing on promoting healthy breakfasts, increasing fruit and vegetable consumption, and improving school meals [43]. Rosenbaum et al. delivered an intervention with the primary aim of reducing risk of Type 2 diabetes in adolescents consisting of health, nutrition and exercise classes, a programme on diabetes risk along with 45-min sessions on Type 2 diabetes prevention in class [58]. The MATCH intervention [41] and the COPE program [53] also used additional physical activity sessions to support the educational interventions. COPE was a manualized 15-session educational and cognitive behavioural skills building programme that also aimed to prevent symptoms of depression in adolescents. The EAT project included school environmental changes such as providing healthier snacks in vending machines, placing educational posters throughout the school, and creating additional play areas [37]. Bogart et al. also used additional food environment modification strategies by providing a variety of fruits and vegetables during school lunch and free water [48]. Campaigns were also conducted to disseminate the messages widely through the schools. Two studies included lunch sessions or breaks to provide healthy foods such as fruit and vegetables [40, 55], and two trialed a modified school nutrition policy [39, 49].

### Meta-analysis

**Fig. 2:**
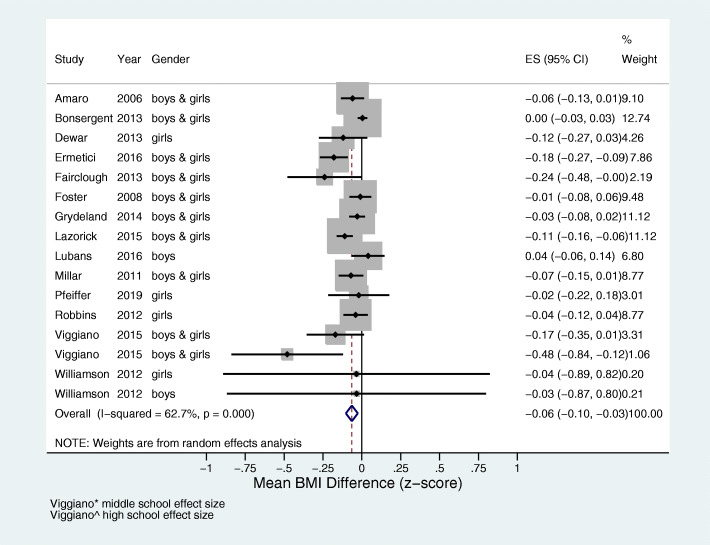
Meta-analysis of 14 studies showing the effect of educational interventions on BMI z-score. The pooled estimate shows significant difference between intervention and control in change in BMI z-score

Of the 33 studies included in the review, 14 studies reporting outcomes based on BMI z-score were included in the meta-analysis [34–47]. There were too few studies that reported effect sizes in a consistent manner to conduct further meta-analyses. There was a high level of heterogeneity between the studies (I^2^ = 62.7%), as seen in Fig. 2. The overall pooled estimate of change in BMI z-score in the intervention group, compared with the control group, was − 0.06, 95% CI (− 0.10, − 0.03); *p* < 0.001.

**Fig. 3:**
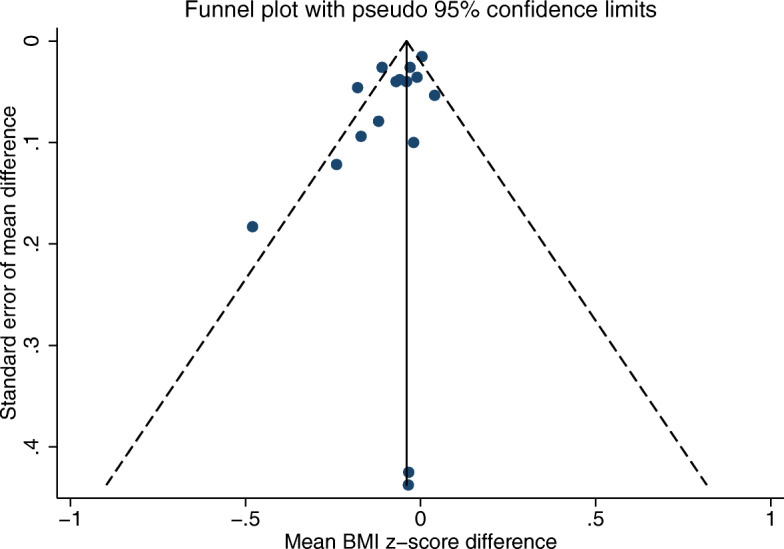
Funnel plot for risk of publication bias

We constructed a funnel plot using the mean difference and standard error of the mean difference in BMI to assess the risk of publication bias (Fig. 3). The asymmetry in the plot indicates a degree of bias within this subset of 14 studies included in the meta-analysis, which might have led to overestimation of effect size. The findings discussed in the meta-analysis and narrative synthesis should therefore be interpreted with caution due to the likelihood of publication bias, high heterogeneity and small effect size.

### Key features of studies with significant effect on BMI outcomes

Overall, fourteen studies reported a significant reduction in BMI and/or BMI z-score [37–41, 43, 45, 48, 49, 52–54, 58, 65]. Of these, four studies reported significant effects in only a subset of the population (See Table 3) [40, 48, 49, 65]. All effective studies with a significant effect on BMI outcomes had a face-to-face component for intervention delivery in the classroom, except one, which was only digital [65]. Of the 16 studies that included parents, eight reported significant effects on BMI outcomes [37, 39, 40, 43, 49, 52–54]. Seven of the effective interventions were RCTs [38–40, 45, 48, 52–54] and seven were non-RCTs [37, 41, 43, 49, 58, 65]. One quasi-experimental study used a capacity-building approach with a community-based component to promote healthy eating and physical activity [43]. There was high variation in the duration of the interventions, from 12 weeks [54] to 3 years [43], with four studies in which the intervention was delivered for a year or more [37, 39, 43, 49]. Even though 14 studies showed significant effect on BMI outcomes, only four studies had a low risk of bias [45, 48, 52, 53]. Five studies had a high risk [39, 43, 49, 58, 65], and the rest a medium risk [37, 38, 40, 41, 54], and this could affect the reliability of the findings. Some studies that were of a low risk of bias did not show significant effects of the intervention on BMI outcomes [36, 42, 44, 47].

Of the 22 interventions delivered by teachers, twelve showed significant effects on BMI outcomes [37–41, 43, 45, 48, 49, 52–54]. Providing CPD/training for teachers prior to the intervention, including some form of face-to-face sessions such as workshops and seminars, was a feature of effective interventions. In the Change! intervention, Fairclough et al. developed the curriculum and resources through formative work with teachers, parents and children [38]. This intervention focused on the interaction between social and environmental factors and their effect on behaviour and provided education on physical activity and nutrition. Subgroup analysis revealing that post-intervention (20 weeks) effects on BMI were significantly greater in girls, but effects on BMI were not sustained at 30 weeks. In the Kaledo study, teachers were trained in how to facilitate and supervise students while playing the game (Kaledo) and there were sustained significant reductions in BMI z-score at 6 and 18-month post-intervention [45]. The game was personalized (participants entered their BMI values) and included a ‘punishment and reward system’ for specific dietary behaviours. It aimed to improve nutrition knowledge and influence dietary habits and eating behaviours of adolescents. Three studies provided more intensive CPD for at least 1 day [39, 41, 53], with one study providing 10 one-hour training sessions across the whole year [39].

Five interventions based on the on social cognitive theory found a statistically significant effect on BMI outcomes [38, 41, 52–54]. The HEALTHY study was a multi-component study which included parents, environmental change, homework activities during breaks and interactive educational lessons in class called ‘FLASH’ delivered by teachers [52]. The module targeted awareness, knowledge, behavioural skills such as goal-setting and peer influence. However, the intervention was delivered in a school with high proportion of ethnic minorities who were at a risk of diabetes (54.2% Hispanic, 18% black). The study led to a non-significant decrease in prevalence of overweight and obesity in both intervention and control schools. Additionally, the mean BMI z-score was significantly lower in the intervention schools than in the control school. The effect of the intervention was higher among students with obesity. However, two multi-component interventions with low risk of bias (NEAT girls and ATLAS) were also based on social cognitive theory and had no short or long-term effects on BMI. These interventions included sports sessions, seminars and nutrition workshops, parent newsletters, pedometers and text messages [36, 42]. Also, of the 16 interventions that included parental involvement, 8 found significant outcomes on BMI [37, 39, 40, 43, 48, 52–54]. The mode of parental involvement in the effective interventions included parent newsletters [52, 53] homework with parents [48], text messages [37], nutrition education and behaviour change for parents [40, 43, 54]. A quasi experimental study [65] led to significant improvement in a subgroup with initial BMI age-adjusted percentile > 50%. Interestingly, there was also a significant increase in BMI age-adjusted percentile for students with BMI percentile less than 50% at baseline. The intervention used methods such as developing peer networks and rewarding good practices to encourage adolescents make healthier choices.

## Discussion

The purpose of this review was to synthesize evidence regarding the effectiveness of school-based health education programmes in reducing BMI and preventing overweight and obesity in adolescents. Overall, we found small but significant reductions in BMI z-score. All but two [58, 65] of the effective interventions were delivered by teachers who were trained prior to the intervention, suggesting that though school-based interventions are often delivered through school-staff, appropriate training/ CPD prior to the intervention could be a crucial component to support the provision and uptake of the intervention. Similarly, many of the effective interventions had included parental involvement and modifications to the school environment. The studies in this review mainly evaluated multi-component interventions that used health education as a tool to improve health behaviours related to diet, physical activity and body composition measures.

Schools are a commonly-used setting for behavioural interventions for preventing obesity and overweight and improving diet and physical activity levels in children and adolescents, as they provide an easy channel for accessing this age group. However, previous reviews of school-based interventions have shown mixed results for BMI outcomes [72, 73]. These reviews suggest that, for children and adolescents, effective interventions targeted direct physical activity and weight reduction through physical education programmes combined with nutrition education. Some studies have shown improvements in the prevalence of overweight and obesity within this age group but only modest effects on BMI [23, 74]. Dietz and Gortmaker (2001) developed a logic model for schools describing the range of factors that influence the energy balance in students [75]. These included a coordinated school health programme that promoted healthy diets and physical activity, school policies and physical education sessions in schools and the surrounding community, along with environmental factors. The results of the present review suggest that schools have the opportunity to effectively deliver evidence-based interventions to prevent obesity.

Various creative methods have been used to engage adolescents in the studies in this review, such as board games [45], digital components (online counselling, SMS messaging) [36, 37, 40, 55, 57, 59, 65, 66] and retreat days [55]. A recent systematic review evaluated the effectiveness of digital interventions in improving diet quality and increasing physical activity in adolescents, suggesting that significant behaviour change can be achieved when health education, goal setting, self-monitoring and parental involvement are included (mainly using web-based platforms, followed by text messages, and games) [76]. Computer-based nutrition education has also led to short-term improvement in BMI [77].

The success of interventions with health education also depends on how the messages are delivered [78]. Complex interventions that are more engaging for adolescents should be developed based on user preferences [79]. Recent RCTs such as the LifeLab intervention have focused on improving adolescents’ understanding of the science behind health messages and motivating them to improve their diet and physical activity levels through hands-on engagement with science [80]. This complex intervention also aims to improve health literacy in adolescents, with preliminary results showing an improvement in their knowledge about risks of NCDs.

It must be noted that many of the studies included in this review were effective in improving other outcomes such as diet, physical activity levels and body fat percentage [34, 37, 44–46, 51, 53, 57, 58]. For example, three studies with no effects on BMI significantly improved levels of moderate to vigorous physical activity [44, 51, 57], and another led to reduced body fat percentage [46]. Four studies with no effect on BMI recruited teenagers with low levels of activity [36, 44, 51, 56] or low cardiorespiratory fitness [56] at baseline, which could have affected the uptake of the intervention. Similarly, some interventions led to a significant effect on BMI for adolescents with obesity at baseline [48, 52, 53]. This could potentially be due to differences in physiological responses to weight loss interventions between adolescents with obesity and those with normal BMI [81]. It should be noted as well that, although we have excluded studies focusing on adolescent eating disorders, future interventions should consider potential unintended effects on body image, eating disorders and other psychological attributes [82].

### Role of stakeholders

Effective interventions often included key stakeholders such as teachers and parents. Previous studies related to other issues in adolescence such as consequences of alcohol consumption have shown that students preferred interventions delivered by teachers [83, 84] and thus the teacher-student relationship can support the effectiveness of school-based interventions. However, these findings should be interpreted with caution. It was not possible to perform a subgroups analysis to determine the role of key stakeholders in the included studies.

Previous systematic reviews in this area reached similar conclusions; that effective physical activity-based interventions, resulting in improved BMI outcomes, were characterized by familial involvement and training for teachers and students on behavioural techniques such as self-monitoring [73]. In this review, most of the effective studies were facilitated by teachers who received CPD in a face-to-face format. Behaviour change frameworks have highlighted the importance of ‘facilitators’ (e.g. qualifications and experience of those delivering the intervention) and ‘pedagogy’ (teaching strategies used by facilitators to deliver the intervention components effectively) in improving intervention engagement and outcomes [85]. A systematic review of teacher CPD in school-based physical activity interventions showed that such programmes were beneficial, particularly when they were conducted for more than 1 day, provided comprehensive pedagogy content, were framed by a theoretical model and measured teachers’ satisfaction with training and content [19]. During data extraction, the reviewers noted that details of teacher CPD are often not elaborated upon or even reported in studies, and hence may have been missed by the present review. The ‘It’s your move!’ project, which used peer-led approaches and capacity-building for teachers, schools and parents highlighted certain challenges such as making time for additional CPD activities along with normal professional commitments for teachers [86]. The authors recommended developing strategies for improving leadership for such complex interventions. Other issues that hinder delivery through teachers in schools include a lack of time or training and uncertainty about their ability and role. Many teachers believed that obesity is a condition that requires treatment [87]. School teachers and personnel often receive little or no training in nutrition or obesity prevention techniques [20, 88, 89]. Providing CPD for teachers for intervention delivery can help them to feel more confident and is part of adopting a health-promoting schools approach that encourages engaging parents and communities [20].

According to the health promoting schools framework of the International Union for Health Promotion and Education (IUHPE), schools have an essential role to play in health education for children and young people [90]. What is less clear is how best to engage with schools and provide evidence-based effective interventions. Our findings suggest that those interventions which showed improvement in BMI outcomes, worked with the school workforce to deliver the intervention. Hence, future interventions could benefit from planning with the education system, and from capitalizing on the expertise of teachers to best deliver messages and engage young people with interventions.

### Strengths and limitations

Other systematic reviews have previously investigated the effectiveness of school-based obesity prevention in children and adolescents; however, this paper is the first to synthesize evidence for BMI outcomes, exclusively in adolescents. Previous reviews explored a broader age range (children and adolescents), different settings (global), or types of interventions (e.g. school and communities). While these reviews helped in providing an overview of the types and effectiveness of interventions for children and teenagers, our review provides in depth information on school-based interventions along with specific recommendations for school stakeholders. The review followed standardized guidance for conducting systematic reviews (full PRISMA checklist in supplementary material) and a rigorous assessment of risk of bias by two independent researchers and reported a detailed narrative synthesis of included studies. A random effects model was used for meta-analysis given the heterogeneous nature of the included studies. Only adjusted results were used for the meta-analysis to reduce bias. Due to the small number of studies eligible for the final meta-analysis, non-RCTs were also included to cover the evidence available. We constructed a funnel plot for the studies included within the meta-analysis. This suggested a degree of publication bias which might have led to over-estimation of effect size within the meta-analysis. The exclusion of studies that considered other anthropometric outcomes such as body fat percentage and waist circumference is an obvious limitation. Though these important anthropometric outcomes predict future risk of NCDs, systematic reviews have shown that the use of BMI to define obesity in children and adolescents is highly specific, albeit with low to moderate sensitivity [91]. To overcome this issue, in the meta-analysis we focused on BMI z-score over absolute BMI or change in BMI, which do not account for adolescent age [92]. Finally, as unpublished analyses, conference proceedings and grey literature were not reviewed, there is a possibility that other school-based interventions were overlooked. Finally, the generalisability of our findings may be limited to high income settings. Although these findings are more directly applicable to interventions developed in school-based settings, the insights will be of use to shape interventions aimed at adolescents.

### Recommendations and implications for research and public health

A detailed analysis of the content of the interventions and comparison based on components was not feasible, as studies often did not include adequate details on these factors in their papers. Future publications of RCTs should consider using standardized ways of reporting intervention details and results, for example using the Template for Intervention Description and Replication (TIDieR) checklist and guide [93]. This improves completeness of reporting for individual study evaluations and further assists reviewers to collate and synthesize the findings. Similarly, future RCTs can consider including mediation analysis of behaviour change components in complex interventions.

Systematic reviews have often been criticized for inadequate consideration of the contexts in which interventions are delivered [94]. Contextual and cultural factors, education systems and prevalence of malnutrition can influence the delivery and uptake of interventions and their effectiveness. Hence, only high-income countries were included in this review. This could have led to omission of recent interventions in LMICs using multi-component behaviour change theories and we recommend that research already conducted in LMICs needs to be reviewed, considering the co-existence of other forms of malnutrition and micronutrient deficiencies with childhood overweight and obesity, to identify the best platforms for interventions in such countries [95]. Similarly, in high-income settings, childhood obesity is often associated with lower SES and poor food environment [96]. While some studies in this review specifically targeted student/ schools from deprived areas [42, 47] future studies need to consider the barriers faced by students from schools in low-income areas who tend to have poorer diets and low physical activity levels use this information to develop targeted interventions. Policies for school health should consider including health education for obesity prevention in personal social health education curriculum, for example, to support the wider public health strategies for obesity prevention. While our review shows that short-term outcomes for BMI can be modified through school-based interventions, further studies need to assess the long-term effects of these interventions and consider the sustainability and implementation at a population level.

## Conclusions

The findings of this systematic review have implications for research and policy in high-income countries, to improve BMI outcomes in adolescence. Overall, our results suggest that school-based health education interventions could potentially help in improving BMI outcomes in the adolescent age group. Interventions should target the biological, psychosocial, environmental and behavioural influences on diet and physical activity. As many of the face-to-face interventions were effective, policy-makers could consider supporting schools to find ways to enable such interventions. Most school-based interventions were delivered by teachers and, including a CPD programme could improve teachers’ confidence in delivering interventions. Alongside schools, parents should be engaged by adopting multi-component strategies to prevent obesity and overweight in adolescents. The research community should facilitate stronger working relationships with and between public health and education teams.

## Supplementary Information

**Additional file 1:.** Search Strategy.

**Additional file 2:.** QUALITY ASSESSMENT RUBRIC.

**Additional file 3:.** PRISMA 2009 Checklist.

## Data Availability

N/A

## Abbreviations

BMI: Body Mass Index
CPD: Continuing Professional Development
CRD: Centre for Reviews and Dissemination
HEIA: Health in Adolescents
LMIC: Low- and Middle-Income Countries
NCD: Non-Communicable Disease
PRISMA: Preferred Reporting Items for Systematic reviews and Meta-Analysis
RCT: Randomised Controlled Trial
SES: Socioeconomic Status
