# Supplementary material for: A systematic review and meta-analysis of school-based interventions with health education to reduce body mass index in adolescents aged 10 to 19 years
Source: Int J Behav Nutr Phys Act. 2021 Jan 4;18:1. doi: 10.1186/s12966-020-01065-9 (PMC7784329; doi:10.1186/s12966-020-01065-9)
Supplement: Supplementary file 1 — Additional file 1:. Search Strategy. [file 12966_2020_1065_MOESM1_ESM.docx]

**Supplementary material:**

1. **Search Strategy**

| **PIO** | **Search terms** |
| --- | --- |
| **Population** | Adolescent/teen/teenager/youth/youths/young people/young person |
| **Intervention** | Health education/ education/ intervention |
| **Outcome** | Body composition/ weight outcomes:  Obesity/obese/weight/ weight loss/ weight reduction/ weight management/weight maintenance/ BMI/ body mass index  Physical activity outcomes:  Physical activity/ exercise/ physical fitness/sports/ sedentary/ screen time/  Diet outcomes:  Die/ vitamins/ vitamin c/ vitamin d/ vitamin b6/dietary fats/ dietary proteins/ dietary carbohydrates/ dietary fibre/ dietary iron/ dietary calcium/ fruit/ vegetables |

**Medline (via ovid)**

| 1 | Adolescent/ |
| --- | --- |
| 2 | adolescen*.mp |
| 3 | (teen or teens or teenager*).mp. |
| 4 | (youth or youths).mp |
| 5 | (young people or young person*).mp |
| 6 | 1 or 2 or 3 or 4 or 5 |
| 7 | physical activity.mp |
| 8 | exp Exercise/ |
| 9 | physical fitness.mp |
| 10 | sports.mp |
| 11 | (sedentary or screen time).mp |
| 12 | **7 or 8 or 9 or 10 or 11** |
| 13 | obes*.mp |
| 14 | overweight.mp |
| 15 | (weight adj2 (loss or losing or lost or reduc* or maintenance or maintain* or manag*)).mp |
| 16 | BMI.mp |
| 17 | body mass index/ |
| 18 | 13 or 14 or 15 or 16 or 17 |
| 19 | 12 or 18 |
| 20 | (health education or education).mp |
| 21 | intervention.mp |
| 22 | 20 or 21 |
| 23 | 6 and 19 and 22 |
| 24 | limit 23 to (english language and humans and yr="2006 - 2020) |
| 25 | exp diet/ |
| 26 | exp vitamins/ |
| 27 | vitamin a/ or vitamin c/ or vitamin d/ or vitamin b6/ |
| 28 | dietary fats/ |
| 29 | dietary proteins/ |
| 30 | dietary carbohydrates/ |
| 31 | dietary fiber/ |
| 32 | dietary iron/ |
| 33 | dietary calcium/ |
| 34 | fruit/ or vegetables/ |
| 35 | 27 or 28 or 29 or 30 or 31 or 32 or 33 or 34 or 35 or 36 |
| 36 | 18 OR 35 |
| 37 | 6 and 22 and 36 |
| 38 | limit 39 to (english language and humans and yr="2006 -Current") |

**Search 24 – citations for studies with focus on Physical activity and Search 38 – studies with focus on dietary interventions were both downloaded sepearately from medline**

**PsychINFO and CINAHL (via EBSCO)**

|  | S36 | S13 AND S21 AND S35 |
| --- | --- | --- |
|  | S35 | S25 OR S26 OR S27 OR S28 OR S29 OR S30 OR S31 OR S32 OR S33 OR S34 |
|  | S34 | fruit OR vegetables |
|  | S33 | "dietary calcium" |
|  | S32 | "dietary iron" |
|  | S31 | "dietary fiber" |
|  | S30 | "dietary carbohydrates" |
|  | S29 | "dietary protein" |
|  | S28 | "dietary fats" |
|  | S27 | (MH "Vitamins+") OR "vitamins" |
|  | S26 | "vitamins" |
|  | S25 | diet |
|  | S24 | S13 AND S21 AND S22 |
|  | S23 | S13 AND S21 AND S22 |
|  | S22 | S14 OR S15 OR S16 OR S17 |
|  | S21 | S18 OR S19 |
|  | S20 | S15 OR S16 OR S17 |
|  | S19 | intervention AND ( health promotion or prevention ) |
|  | S18 | education |
|  | S17 | obes' or overweight or body mass index or bmi or weight |
|  | S16 | "screen time" |
|  | S15 | sedentary |
|  | S14 | physical activity or exercise or fitness or sport |
|  | S13 | adolescen' or juvenil' or teen' or youth or young people or young person |
|  | S12 | S1 AND S9 AND S10 |
|  | S11 | S1 AND S9 AND S10 |
|  | S10 | S2 OR S3 OR S4 OR S5 |
|  | S9 | S6 OR S7 |
|  | S8 | S3 OR S4 OR S5 |
|  | S7 | intervention AND ( health promotion or prevention ) |
|  | S6 | education |
|  | S5 | obes' or overweight or body mass index or bmi or weight |
|  | S4 | "screen time" |
|  | S3 | sedentary |
|  | S2 | physical activity or exercise or fitness or sport |
|  | S1 | adolescen' or juvenil' or teen' or youth or young people or young person |

**ERIC**

((adolescen* OR teen* OR youth* OR "young people" OR "young person*") AND ("physical activity" OR exercise OR "physical fitness" OR sports OR sedentary OR "screen time" OR obes* OR overweight OR "weight loss" OR "weight management" OR "body mass index") AND ("health education" OR education))

AND stype.exact("Scholarly Journals") AND la.exact("English") AND pd(20060101-20161101)

**Limited by:**

Date: From January 01 2006 to June 2020

Document type:

53 types searched

Source type:

Scholarly Journals

Language: English
