# Supplementary material for: A systematic review and meta-analysis of school-based interventions with health education to reduce body mass index in adolescents aged 10 to 19 years
Source: Int J Behav Nutr Phys Act. 2021 Jan 4;18:1. doi: 10.1186/s12966-020-01065-9 (PMC7784329; doi:10.1186/s12966-020-01065-9)
Supplement: Supplementary file 2 — Additional file 2:. QUALITY ASSESSMENT RUBRIC. [file 12966_2020_1065_MOESM2_ESM.docx]

**Supplementary material 2: QUALITY ASSESSMENT RUBRIC**

|  | Item | Risk of bias | | |
| --- | --- | --- | --- | --- |
|  |  | Low (+1) | Medium (0) | High (-1) |
| 1 | Was the study design appropriate? | Randomised controlled trial | Quasi-experimental studies that include a control group | Experimental studies that do not use a control group. |
| 2 | Was assignment to treatment groups truly random? | Random numbers table or computer randomisation | Randomised by methods other than random number table, or randomised in large clusters. | Not randomised, or inadequate randomisation methods such as birth date used. OR randomisation methods not described |
| 3 | Were outcome assessors blinded to the treatment allocation? | Assessors (statisticians, anyone taking measurements, and the participants in self-reported questionnaires) are unaware of treatment allocation. |  | At least one assessor is aware of the treatment allocation OR blinding not discussed |
| 4 | Were participants blinded to the treatment allocation? | A good control was used so that participants were not aware whether they were receiving the intervention. | Procedures state that a control was used, but provides little detail OR control is not totally comparable to intervention | No control used |
| 5 | Were groups similar at baseline? | Appropriate statistical tests (chi-square and/or t-tests) used to analyse differences between groups at baseline, and found that there were no significant differences. | Groups are similar, but there are some differences that are judged to be acceptable. | Analyst did not test for differences at baseline, or there were significant differences between groups. |
| 6 | Were selection criteria adequately specified? | Selection criteria are very clear. | Selection criteria reported, but not very specific. | Selection criteria are not reported. |
| 7 | What was the approach to selection of participants? | Participants were randomly selected. | School based interventions that require pupil/parental consent. | Volunteers are recruited. |
| 8 | What proportion of participants were lost to follow-up? | Loss to follow up of less than 10% AND similar loss in all groups. | More drop outs than expected (10%-30%). Similar loss between groups. | High dropout rate (>30%) and/or large difference in follow up between groups. |
| 9 | BMI, BMI- Z Score Assessment | Reliable and validated measures  Measured by trained staff | Not reported | Self-reported |
| 10 | Was the intervention executed in the same way across all participants/ clusters? | The intervention was delivered in a way that was not variable eg. Computer-generated. | The intervention was probably delivered consistently. Eg. The same person delivering a lesson to multiple groups. | The intervention was likely have been delivered differently across the trial. Eg. Asking teachers to add elements to their curriculum without further training. |
| 11 | Were point estimates and measures of variability reported for primary outcome measures? | All outcomes are reported with measures of variability such as SD, SE, or CI | Some variability measures are reported | No variability measures are reported |
| 12 | Did the analysis include an intention to treat analysis? | Intention to treat analysis was clearly used |  | Intention to treat was not used, or not stated |
| 13 | Were the analytical methods appropriate? | Statistical tests used are rigorous, and appropriate for the data set. (Regression modelling, ANOVA, etc.) | Statistical methods are effective, but not as rigorous as they could be. | Statistical methods are limited, and only report descriptive stats OR tests used are not appropriate for the dataset. |
| 14 | Did the analysis adjust for confounding?   - Educational attainment or SES - Gender - Age - Baseline behaviour - School cluster | The statistical model used adjusts for all relevant confounding factors. | The model adjusts for 3 to 4 confounders. | Does not report adjusted results or < 3 factors |
| 15 | Was the sample size appropriate? | Power calculation is reported, and sample size meets the requirements of the power calculation. | Power calculation is not reported, but text states that recruitment was based on a power calculation OR sample size is very large (>1000) | No power calculation, unclear whether sample size is adequate OR powered for measure other than dietary behaviour |
